# Association of maternal vitamin B_12_ and folate levels in early pregnancy with gestational diabetes: a prospective UK cohort study (PRiDE study)

**DOI:** 10.1007/s00125-021-05510-7

**Published:** 2021-07-22

**Authors:** Ponnusamy Saravanan, Nithya Sukumar, Antonysunil Adaikalakoteswari, Ilona Goljan, Hema Venkataraman, Amitha Gopinath, Christos Bagias, Chittaranjan S. Yajnik, Nigel Stallard, Yonas Ghebremichael-Weldeselassie, Caroline H. D. Fall

**Affiliations:** 1 Division of Health Sciences, Warwick Medical School, Gibbet Hill, University of Warwick, Warwick, Coventry, UK; 2 Academic Department of Diabetes, Endocrinology and Metabolism, George Eliot Hospital, Nuneaton, UK; 3 Department of Biosciences, School of Science and Technology, Nottingham Trent University, Clifton, Nottingham, UK; 4 Novo Nordisk Ltd, Gatwick, UK; 5 Heartlands Hospital, University Hospital Birmingham NHS Trust, Birmingham, UK; 6 Diabetes Unit, KEM Hospital & Research Centre, Pune, India; 7 School of Mathematics and Statistics, The Open University, Milton Keynes, UK; 8 MRC Lifecourse Epidemiology Unit, University of Southampton, Southampton, UK

**Keywords:** Folate, Folic acid, Gestational diabetes mellitus, Micronutrients, Pregnancy, Risk factors, Vitamin B_12_

## Abstract

**Aims/hypothesis:**

The prevalence of gestational diabetes mellitus (GDM) is increasing worldwide in all ethnic groups. Low vitamin B_12_ and low/high folate levels may contribute to GDM risk, but there is conflicting evidence. Our aim is to assess the relationships of early pregnancy vitamin B_12_ and folate levels with the risk of GDM status at 26–28 weeks of gestation.

**Methods:**

This was a prospective, multi-centre, multi-ethnic cohort study (*n* = 4746) in the UK. Participants who were eligible to be selectively screened as per the National Institute for Health and Care Excellence (NICE) criteria were included in the study.

**Results:**

GDM prevalence was 12.5% by NICE and 14.7% by International Association of Diabetes and Pregnancy Study Groups (IADPSG) criteria. Folate deficiency (1.3%) was rare but B_12_ insufficiency (42.3% at <220 pmol/l) and folate excess (36.5%) were common in early pregnancy. Early pregnancy median B_12_ levels were lower, and folate levels higher, in women who were diagnosed with GDM at 26–28 weeks. B_12_ was negatively associated with fasting plasma glucose (1 SD: −0.06 mmol/l; 95% CI −0.04, −0.08; *p* < 0.0001) and 2 h plasma glucose levels (−0.07 mmol/l; 95% CI −0.02, −0.12; *p* = 0.004). Higher B_12_ was associated with 14.4% lower RR of IADPSG-GDM (0.856; 95% CI 0.786, 0.933; *p* = 0.0004) after adjusting for key confounders (age, parity, smoking status, ethnicity, family history, household income and folate status). Approximately half of this association was mediated through BMI. Folate was positively associated with 2 h plasma glucose levels (0.08 mmol/l; 95% CI 0.04, 0.13; *p* = 0.0005) but its relationship with fasting plasma glucose was U-shaped (quadratic β: 0.011; *p* = 0.05). Higher folate was associated with 11% higher RR of IADPSG-GDM (adjusted RR 1.11; 95% CI 1.036, 1.182; *p* = 0.002) (age, parity, smoking status, ethnicity, family history, household income and B_12_ status). Although no interactions were observed for B_12_ and folate (as continuous variables) with glucose levels and GDM risk, a low B_12_–high folate combination was associated with higher blood glucose level and risk of IADPSG-GDM (adjusted RR 1.742; 95% CI 1.226, 2.437; *p* = 0.003).

**Conclusions/interpretation:**

B_12_ insufficiency and folate excess were common in early pregnancy. Low B_12_ and high folate levels in early pregnancy were associated with small but statistically significant changes in maternal blood glucose level and higher RR of GDM. Our findings warrant additional studies on the role of unmetabolised folic acid in glucose metabolism and investigating the effect of optimising early pregnancy or pre-conception B_12_ and folate levels on subsequent hyperglycaemia.

**Trial registration:**

ClinicalTrials.gov NCT03008824.

**Graphical abstract:**

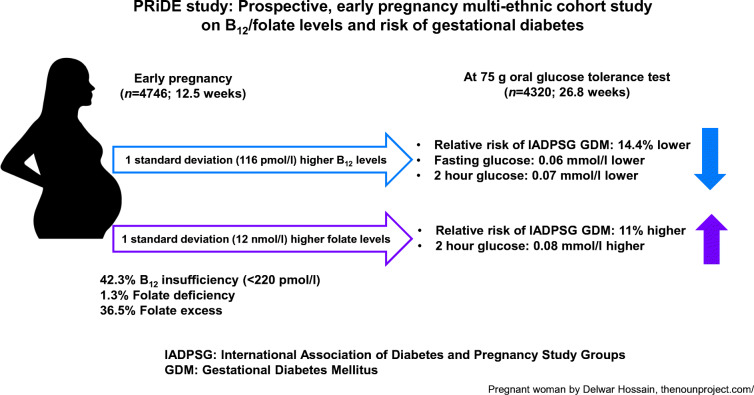

**Supplementary Information:**

The online version contains peer-reviewed but unedited supplementary material available at 10.1007/s00125-021-05510-7.

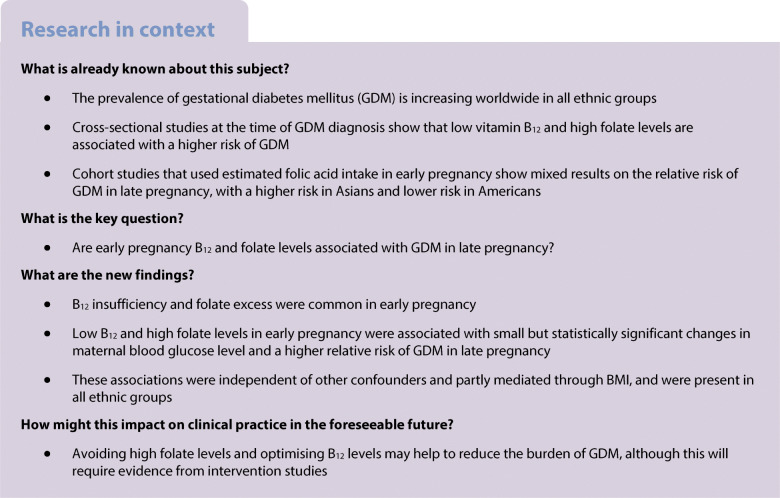



## Introduction

Gestational diabetes mellitus (GDM) is a common medical disorder in pregnancy, estimated to affect more than 20 million pregnancies worldwide, and causes significant short- and long-term consequences to both the women and their offspring [1]. While management of GDM reduces the short-term complications, it does not abolish them completely [2, 3]. In addition, adverse effects for the offspring may have already happened prior to the diagnosis of GDM [4–6]. Therefore, prevention of GDM would be a better approach to reduce adverse outcomes in pregnant women and their offspring. This will require identifying and modifying risk factors before the diagnosis of GDM. However, most prevention studies in early pregnancy (such as diet and lifestyle interventions) have focused on a single modifiable risk factor, obesity, with limited success. Identification of other modifiable risk factors that contribute to GDM risk is needed.

Folate and B_12_ are essential micronutrients for the metabolism of single carbon atoms (known as 1-C metabolism) and these pathways are involved in DNA methylation and synthesis of amino acids, nucleic acids and lipids [7, 8]. High total homocysteine (tHcy) is a marker of deficiency of either nutrient as B_12_ is a coenzyme for the folate-dependent methylation of tHcy to methionine. In addition, B_12_ is a coenzyme for the action of methyl malonyl-CoA mutase in the mitochondria, involved in the conversion of methyl malonyl-CoA to succinyl-CoA, and degradation of odd-chain fatty acids and branched-chain amino acids (BCAAs) [7, 8]. Altered levels of BCAAs have been shown to precede the onset of hyperglycaemia and type 2 diabetes [9]. Folate and B_12_ thus play a crucial and inter-dependent role in genomic stability, methylation potential, epigenetics, carbohydrate metabolism, fatty acid β-oxidation and protein synthesis [7, 8]. Some of these mechanisms contribute to glucose homeostasis as well as adverse metabolic programming of the offspring [10–12].

Conflicting data have been reported regarding associations of B_12_ and folate with the risk of GDM. Most of the studies measured these B vitamins at the time of diagnosis of GDM in late pregnancy or used estimated folate intake [13–21]. Higher tHcy has been linked to impaired endothelial function, with resulting reduction in insulin sensitivity, outside pregnancy [22], but its role in glucose metabolism in pregnancy is not clear. Previous studies showed positive, negative or no associations with the risk of GDM [23–26]. The purpose of our study is to examine the relationships of B_12_, folate and tHcy levels in early pregnancy with the glucose levels and risk of GDM in late pregnancy, in a large, multi-ethnic, prospective cohort study.

## Methods

### Participant selection and recruitment

Pregnant women attending antenatal care in ten study sites across the UK were recruited between 2012 and 2018 to the ‘Micronutrients in Pregnancy as a Risk Factor for Gestational Diabetes and Effects on Mother and Baby’ (PRiDE) study (clinicaltrials.gov number: NCT03008824). Ethical approval was obtained from the National Research Ethics Committee (12/WM/0010). All participants provided written informed consent. The detailed study protocol, including all the standard operating procedures, is available at the departmental website, University of Warwick (https://warwick.ac.uk/fac/sci/med/staff/saravanan/pride_study_protocol.pdf). In brief, pregnant women aged between 18 and 45 years who were at less than 16 weeks of gestation and fulfilled the National Institute for Health and Care Excellence (NICE) criteria for screening for GDM were included [27]. All centres followed the NICE screening guidelines (at least one of the following risk factors): BMI ≥30 kg/m^2^; previous GDM; previous unexplained stillbirth or birthweight ≥4.5 kg; first degree relative with diabetes; ethnic minority group. Two centres screened additional women who were aged ≥35 years at booking or had a history of polycystic ovarian syndrome. The exclusion criteria were: pre-gestational diabetes mellitus; a previous pregnancy with a neural tube defect; multiple gestation; severe anaemia (haemoglobin <10 g/dl); confirmed vitamin B_12_ or folate deficiency or having received B_12_ injections within the last 6 months.

### Data collection

Participants’ demographic information including their marital, employment and smoking status; educational attainment; household income; and medical, obstetric and supplement intake (type and duration) history were collected during the screening visit. Their height, weight, BMI and waist circumference were measured at the booking and OGTT visits. A random blood sample at booking and fasting and 2 h blood samples at OGTT using 75 g of anhydrous glucose after an overnight fast of at least 10 h were taken. As the focus of this analysis is the diagnosis of GDM, birth outcomes data are not reported here.

### Biochemical analysis

Blood samples were kept in refrigerated containers, centrifuged within 30 min of collection and stored in −80°C freezers until analysis, except for glucose which was done immediately. Plasma glucose was determined by the hexokinase enzymatic method using a Synchron CX7 auto-analyser (Beckman Coulter, Fullerton, CA, USA). Serum B_12_ and folate were measured by electro-chemiluminescent immunoassay (Roche Cobas analyser, Roche Diagnostics, Burgess Hill, UK). The intra- and inter-assay CVs for B_12_ and folate were 2.0% and 3.1%; and 3.1% and 3.8%, respectively. Plasma tHcy was determined by stable isotopic dilution analysis using a Shimadzu HPLC system with an auto-sampler coupled to the detection system of an API 6500 QTrap tandem mass spectrometer (liquid chromatography mass spectrometry [LCMS]) (Applied Biosystems, Warrington, UK) [28]. A calibration curve and quality control samples (low, medium and high concentrations within the limits of quantification; Waters, UK) were set up for each sample batch that was analysed. Inter- and intra-assay CVs for tHcy were 7.0% and 8.1%. This was comparable to an assay used by a regional laboratory in Birmingham [29] and below the recommended CV thresholds [30].

### Definitions

Ethnicity was coded using standard definitions. ‘Other’ ethnic group included North African, Black African, Caribbean, Asian, South East Asian, Middle Eastern and mixed ethnicity. GDM diagnoses based on NICE (NICE-GDM; fasting plasma glucose [FPG] ≥5.6 mmol/l or 2 h post-load plasma glucose [2 h-PG] ≥7.8 mmol/l) and International Association of Diabetes and Pregnancy Study Groups (IADPSG) criteria (IADPSG-GDM; FPG ≥5.1 mmol/l or 2 h-PG ≥8.5 mmol/l) were recorded [27, 31]. B_12_ insufficiency was defined using two different cut-offs: <150 pmol/l and <220 pmol/l. The former is commonly used to define B_12_ deficiency in adults and in pregnancy, while others have suggested the higher cut-off in pregnancy, as this was associated with low birthweight and elevated tHcy and methylmalonic acid (MMA) levels [32–34]. Folate deficiency was defined as <10 nmol/l and folate excess as >45 nmol/l [35].

### Pre-defined outcomes

#### Primary

Differences in the risk of GDM in women with and without early pregnancy B_12_ insufficiency.

#### Secondary

1. Associations of B_12_ and folate with blood glucose level/GDM risk (B_12_, folate, FPG and 2h-PG as continuous variables);
2. Association between tHcy and blood glucose level/GDM risk;
3. Association between ‘low B_12_–high folate’ status and blood glucose level/GDM risk; and
4. Ethnic differences in associations of B_12_, folate and tHcy with blood glucose level/GDM risk.
5. We also aimed to assess the role of BMI in these associations.

### Statistical analysis

#### Sample size calculations

There are no published data on the rate of first trimester B_12_ insufficiency in GDM women in the UK. At study design, preliminary results from our group showed 15% insufficiency (<150 pmol/l) in early pregnancy and 10–12% prevalence of GDM [27]. To detect a 5% difference in the prevalence of GDM between normal and B_12_ insufficiency with 90% power at the 5% significance level, 3822–4322 women were required; we aimed to recruit 4500 women in early pregnancy, allowing for a 10% drop-out rate.

#### Analyses

Women’s characteristics were reported using appropriate descriptive statistics (mean and SD) or median and IQR for continuous variables and percentages for categorical variables. χ^2^ and *t* tests were used to assess differences in categorical and continuous variables, respectively. To define participants’ socioeconomic status (SES), we considered their occupation and total household income. For the main outcome analyses, household income was used as the main measure of SES, because this is directly associated with higher purchase and intake of vegetables and fruit which in turn correlate with higher folate status [36].

RR and 95% CI for GDM in relation to different micronutrients were estimated using multiple log-binomial regression models. Multiple linear regression analyses were used to test the associations of early pregnancy micronutrients with FPG and 2h-PG at OGTT. The micronutrients were standardised, by subtracting the mean and dividing by SD, to aid comparison. To further understand the shapes of the relationships of FPG and 2h-PG at OGTT with B_12_ and folate, cubic spline regression models were fitted. Ethnic-specific associations of the micronutrients with glucose levels and the risk of GDM were tested using similar regression models.

From published studies, while the causal direction is clear between BMI and glucose, this is not clear between BMI and B_12_ and folate. Therefore, to understand this complex interplay, two different models were used, a priori, for all the regression analyses while adjusting for possible confounders. Model 1 included age, parity, smoking status, ethnicity, family history, household income and respective micronutrient status (B_12_ for folate, folate for B_12_, and B_12_ and folate for tHcy). Model 2 included model 1 plus BMI. Other possible confounders such as marital status and gestational weight gain were not used as they did not contribute to the exploratory and/or outcome variables. Effects of interaction between B_12_ and folate on blood glucose level and the risk of GDM were investigated both as continuous variables and after categorising them by tertiles. The first tertile of folate and the third tertile of B_12_ were used as the reference tertiles.

As the proportion of missing data was low for B_12_, folate and tHcy (maximum 2.6%), the main analysis reported excluded these patients. All analyses were implemented using R version 4.0.0 [37].

## Results

**Fig. 1:**
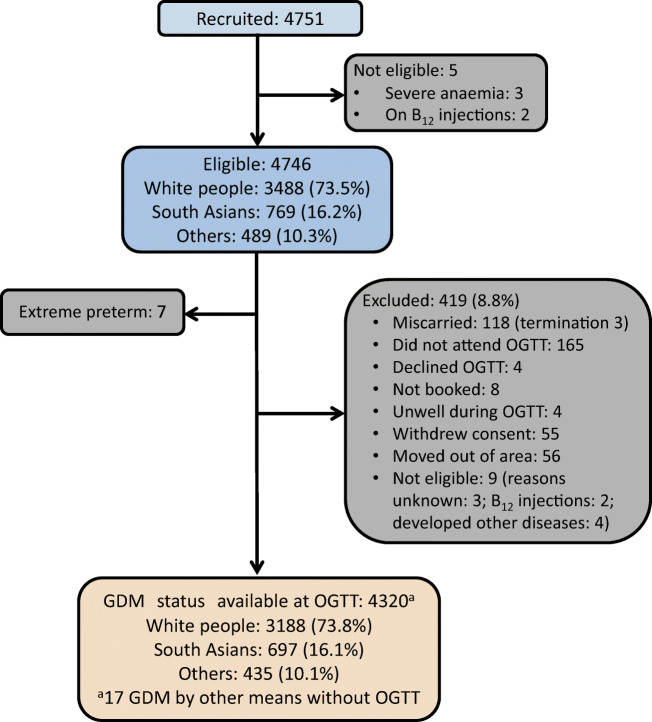
Study flow diagram

A total of 4746 eligible pregnant women were recruited. Their mean gestational age was 12.5 ± 1.4 weeks (Fig. 1). Ethnicity proportions were similar at both the booking and OGTT visits (Table 1, Fig. 1). B_12_ insufficiency (42.3%) and folate excess (36.5%) were common but folate deficiency (1.3%) was rare in all ethnic groups. BMI was inversely associated with B_12_ and positively associated with folate and tHcy (electronic supplementary material [ESM] Table 1). Folic acid and multivitamin supplement usage were associated with higher folate and B_12_ levels, respectively, and lower tHcy. While household income was unrelated to B_12_ levels, higher income was associated with higher folate and lower tHcy levels.

**Table 1:** Baseline characteristics of the study participants

Maternal characteristic at booking	All (*N* = 4746)	White people (*n* = 3488)	South Asians (*n* = 769)	Other (*n* = 489)	*p* value
Age (years)	30.51 ± 5.29	30.1 ± 5.35	31.57 ± 4.72	31.4 ± 5.4	<0.0001
Multiparity (≥2)	919 (19.4)	698 (20.0)	119 (15.5)	102 (20.9)	0.01
Gestational age (weeks)	12.45 ± 1.44	12.39 ± 1.47	12.51 ± 1.39	12.8 ± 1.21	<0.0001
Height (cm)	164.28 ± 6.83	165.32 ± 6.64	160.69 ± 6.24	162.57 ± 6.84	<0.0001
Weight (kg)	83.33 ± 20.49	88.3 ± 19.6	66.44 ± 13.93	74.44 ± 18.04	<0.0001
BMI (kg/m^2^)	30.8 ± 7.06	32.3 ± 6.92	25.7 ± 4.96	28.1 ± 6.27	<0.0001
Waist circumference (cm)^a^	98.54 ± 16.41	101.9 ± 15.95	87.73 ± 12.78	91.45 ± 15.16	<0.0001
Biochemical characteristics	*n* = 4630	*n* = 3396	*n* = 759	*n* = 475	
B_12_ (pmol/l)	238.2 (183.3–311.2)	230.3 (181.1–298.0)	233.3 (177.9–319.9)	316.5 (237.3–429.3)	<0.0001
Folate (nmol/l)	35.9 (24.8–52.2)	33.8 (23.2–51.3)	43.0 (30.4–54.0)	38.3 (27.0–51.6)	<0.0001
tHcy (μmol/l)^b^	11.3 (8.6–14.7)	11.4 (8.7–14.8)	11.1 (8.7–14.7)	10.9 (8.3–13.8)	0.12
B_12_ insufficiency at <150 pmol/l	490 (10.6)	365 (10.7)	101 (13.3)	23 (4.8)	<0.0001
B_12_ insufficiency at <220 pmol/l	1985 (42.8)	1536 (45.2)	348 (45.8)	98 (20.6)	<0.0001
Folate deficiency (<10 nmol/l)	69 (1.5)	61 (1.8)	1 (0.1)	7 (1.5)	0.003
Folate excess (>45 nmol/l)	1640 (35.4)	1139 (33.5)	342 (45.1)	159 (33.5)	<0.0001
Using folate supplements	3424 (78.2)	2569 (79.5)	526 (76.0)	329 (72.3)	<0.0001
Using multivitamin supplements	2494 (58.1)	1840 (58.2)	425 (62.7)	229 (50.9)	<0.0001
Maternal characteristics at OGTT	*n* = 4320	*n* = 3396	*n* = 759	*n* = 475	
Gestational age (weeks)^c^	26.79 ± 2.64	26.83 ± 2.63	26.6 ± 2.68	26.76 ± 2.67	0.12
Gestational weight gain (kg)^d^	5.86 ± 4.75	5.65 ± 4.85	6.59 ± 4.7	6.29 ± 3.85	<0.0001
FPG (mmol/l)^e^	4.48 ± 0.55	4.49 ± 0.54	4.51 ± 0.56	4.4 ± 0.56	0.002
2h-PG (mmol/l)^f^	5.88 ± 1.51	5.85 ± 1.47	6.03 ± 1.63	5.8 ± 1.59	0.01
GDM prevalence by NICE^g^	538 (12.5)	390 (12.2)	95 (13.6)	53 (12.2)	0.59
GDM prevalence by IADPSG^g^	633 (14.7)	450 (14.1)	127 (18.2)	57 (13.1)	0.01

Data are presented as mean ± SD, median (IQR) or *n* (%)

‘Other’ ethnic group includes: North African, Black African, Caribbean, Asian, South East Asian, Middle Eastern and mixed ethnicity

*p* values across all ethnic groups by χ^2^ test

Variables that have lesser ‘*n*’ are indicated separately:

^a^All = 4366, white people = 3213, South Asian = 696, other = 457

^b^All = 4612, white people = 3385, South Asian = 753, other = 474

^c^All = 4235, white people = 3125, South Asian = 680, other = 430

^d^All = 3974, white people = 2953, South Asian = 629, other = 392

^e^All = 4303, white people = 3172, South Asian = 696, other = 435

^f^All = 4275, white people = 3150, South Asian = 692, other = 433

^g^All = 4320, white people = 3188, South Asian = 697, other = 435

Obesity was the most common reason for selective screening in white women, and all ethnic minority women were universally screened as per NICE guidelines (ESM Table 2). A total of 538 (12.5%) women had NICE-GDM and 633 (14.7%) had IADPSG-GDM. The characteristics of study participants by GDM status are shown in Table 2 (IADPSG-GDM). For simplicity, all the NICE-GDM data are presented in the ESM (ESM Table 3, ESM Fig. 1). Both folic acid and multivitamin supplement usage were similar between GDM and non-GDM groups.

**Table 2:** Characteristics of study participants by IADPSG-GDM

Maternal characteristic	All (*N* = 4320)	GDM (*n* = 633)	Non-GDM (*n* = 3687)	*p* value
Ethnicity				
White people	3188 (73.8)	449 (14.1)	2739 (85.9)	0.01
South Asian	697 (16.1)	127 (18.2)	570 (81.8)	
Other	435 (10.1)	57 (13.1)	378 (86.9)	
Age (years)	30.59 ± 5.23	31.9 ± 5.16	30.36 ± 5.21	<0.0001
Multiparity (≥2)	806 (18.7)	137 (21.6)	669 (18.1)	0.04
Gestational age at booking (weeks)	12.47 ± 1.41	12.36 ± 1.46	12.49 ± 1.4	0.04
Gestational age at OGTT (weeks)^a^	26.79 ± 2.64	26.07 ± 3.67	26.91 ± 2.4	<0.0001
Height (cm)	164.33 ± 6.85	164.08 ± 6.84	164.37 ± 6.85	0.31
Weight (kg)	83.54 ± 20.46	89.25 ± 20.46	82.56 ± 20.3	<0.0001
BMI (kg/m^2^)	30.86 ± 7.07	33.05 ± 6.87	30.49 ± 7.03	<0.0001
Waist circumference (cm)^b^	98.57 ± 16.35	103.98 ± 15.99	97.64 ± 16.23	<0.0001
Gestational weight gain (kg)^c^	5.86 ± 4.75	5.28 ± 4.45	5.95 ± 4.79	0.001
Using folate supplements^d^	3136 (78.5)	482 (81.6)	2654 (78)	0.06
Using multivitamin supplements^e^	2317 (59.2)	331 (58.3)	1986 (59.4)	0.65
Biochemical characteristics	*n* = 4228	*n* = 617	*n* = 3611	
B_12_ (pmol/l)	239.1 (183.76–313.3)	223.7 (179.0–300.3)	242.0 (185.4–315.3)	0.0007
Folate (nmol/l)	36.7 (25.5–52.7)	38.2 (26.9–61.7)	36.2 (25.1–52.1)	0.05
tHcy (μmol/l)^f^	11.2 (8.6–14.6)	10.9 (8.1–13.9)	11.4 (8.7–14.8)	0.02
B_12_ insufficiency at <150 pmol/l	436 (10.3)	71 (11.5)	365 (10.1)	0.32
B_12_ insufficiency at <220 pmol/l	1790 (42.3)	298 (48.3)	1492 (41.3)	0.001
Folate deficiency (<10 nmol/l)	54 (1.3)	9 (1.5)	45 (1.2)	0.81
Folate excess (>45 nmol/l)	1544 (36.5)	240 (38.9)	1304 (36.1)	0.20

Data are presented as mean ± SD, median (IQR) or *n* (%)

‘Other’ ethnic group includes: North African, Black African, Caribbean, Asian, South East Asian, Middle Eastern and mixed ethnicity

*t* test was used to compare the continuous variables between GDM and non-GDM. χ^2^ test was used to compare the categorical variables (ethnicity, multiparity, B_12_ insufficiency, folate deficiency and folate excess)

Variables that have lesser ‘*n*’ are indicated separately:

^a^All = 4227, GDM = 607, non-GDM = 3620

^b^All = 3979, GDM = 583, non-GDM = 3396

^c^All = 3968, GDM = 556, non-GDM = 3412

^d^All = 3994, GDM = 591, non-GDM = 3403

^e^All = 3912, GDM = 568, non-GDM = 3344

^f^All = 4209, GDM = 613, non-GDM = 3596

### Primary outcome: B_12_ insufficiency and GDM

Early pregnancy median B_12_ levels were significantly lower in women who were later diagnosed to have IADPSG-GDM (Table 2). B_12_ insufficiency at <220 pmol/l was associated with 38.3% higher adjusted RR (aRR) of IADPSG-GDM (aRR 1.383; 95% CI 1.157, 1.652; *p* = 0.0004) in model 1 and 20.3% higher in model 2 (aRR 1.203; 95% CI 1.003, 1.443; *p* = 0.05). The associations with NICE-GDM and with B_12_ insufficiency at <150 pmol/l are shown in ESM Table 4.

### Secondary outcomes

#### B_12_, blood glucose level and GDM

**Fig. 2:**
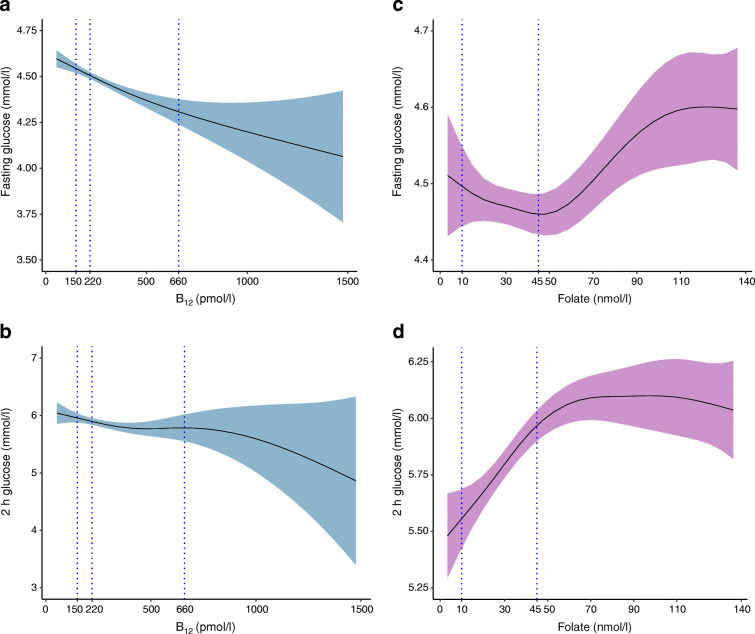
(**a**–**d**) Cubic spline regression analysis of the relationship of early pregnancy B_12_ and folate with fasting and 2 h glucose levels at OGTT. (**a**) The relationship between B_12_ and fasting glucose and (**b**) 2 h glucose and (**c**) the relationship between folate and fasting glucose and (**d**) 2 h glucose using the cubic spline regression analyses. The regression curve is shown in bold, and the shaded area represents the 95% confidence band. The dotted vertical lines represent the reference range for the B_12_ (150, 220 and 660 pmol/l) and folate levels (10 and 45 nmol/l)

**Fig. 3:**
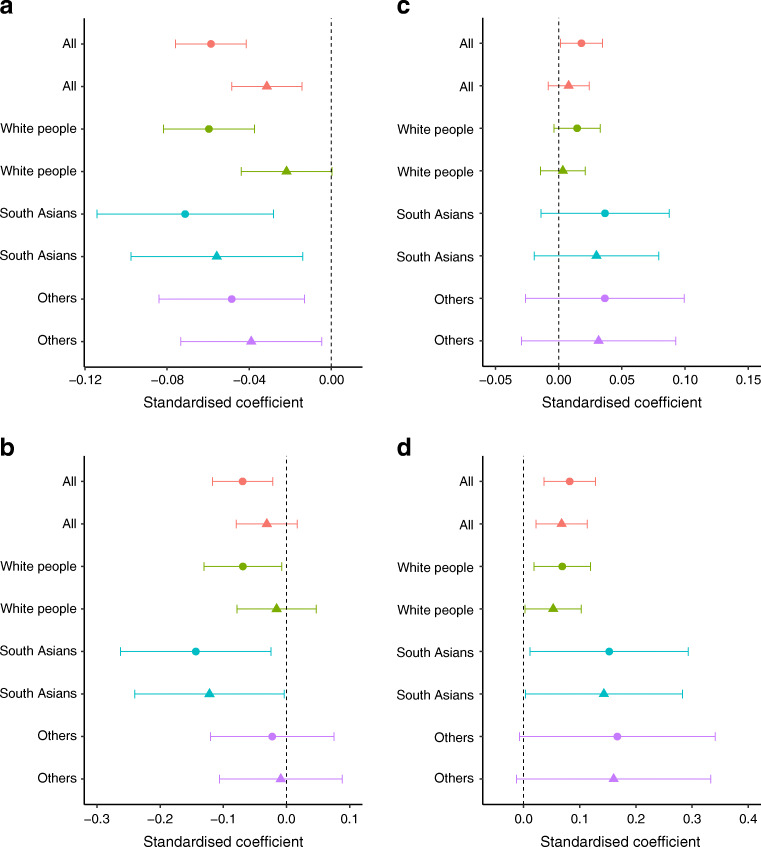
(**a**–**d**) Associations of early pregnancy B_12_/folate levels with fasting and 2 h glucose levels. (**a**) The standardised β coefficient of the associations of early pregnancy B_12_ levels (as a continuum) with fasting glucose at OGTT and (**b**) with 2 h glucose levels. (**c**) The standardised β coefficient of the associations of early pregnancy folate levels (as a continuum) with fasting glucose at OGTT and (**d**) with 2 h glucose levels. Data are shown with 95% CI. Two models are shown for each ethnic group. Circles depict model 1 and triangles depict model 2. Model 1 is adjusted for the following covariates: age, parity, family history, household income, smoking and folate. Model 2 is adjusted for model 1 + BMI

**Fig. 4:**
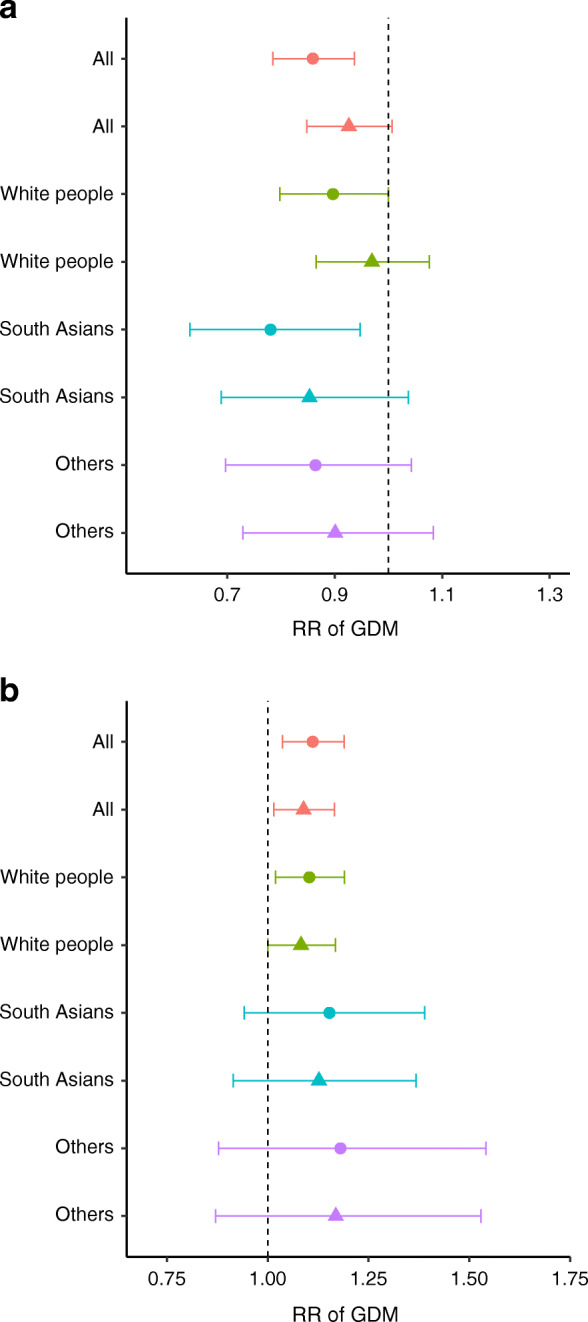
(**a**, **b**) Early pregnancy B_12_/folate levels and risk of GDM. (**a**) The B_12_ and (**b**) folate levels in early pregnancy and aRR of IADPSG-GDM. Data are shown with 95% CI. Two models are shown for each ethnic group. Circles depict model 1 and triangles depict model 2. Model 1 is adjusted for the following covariates: age, parity, family history, household income, smoking and B_12_. Model 2 is adjusted for model 1 + BMI

The cubic spline graphs showed that the relationships between B_12_ and glucose levels were linear (Fig. 2a,b) and B_12_ was negatively associated with FPG, 2h-PG and risk of GDM (Figs 3a,b, 4a). A 1 SD increase in B_12_ (116 pmol/l) was associated with a 0.06 mmol/l lower FPG (95% CI −0.04, −0.08; *p* < 0.0001), a 0.07 mmol/l lower 2h-PG (95% CI −0.02, −0.12; *p* = 0.004) and a 14.4% lower RR of IADPSG-GDM (0.856; 95% CI 0.786, 0.933; *p* = 0.0004; model 1), after adjusting for key confounders. Effect sizes halved when adjusted for BMI (model 2; Figs 3a,b, 4a).

#### Folate, blood glucose level and GDM

Folate was positively associated with blood glucose level, but its associations were complex. The cubic spline graphs show the different shapes of these relationships. With FPG, the relationship was U-shaped (quadratic β: 0.011; *p* = 0.05) and with 2h-PG it was linear and significant (Fig. 2c,d). A 1 SD increase in folate (12.16 nmol/l) was associated with 0.08 mmol/l higher 2 h-PG (95% CI 0.04, 0.13; *p* = 0.0005) and 11% higher RR of IADPSG-GDM (aRR 1.11; 95% CI 1.036, 1.182; *p* = 0.002) (model 1; Figs 3c,d, 4b). Similar to B_12_, its effect size reduced when adjusted for BMI (model 2; Figs 3c,d, 4b). A sensitivity analysis, stratified by whether women were taking folate supplements or not, showed similar results (data not shown).

#### tHcy, blood glucose level and GDM

tHcy was inversely associated with blood glucose level and risk of GDM. The associations strengthened when adjusted for BMI, suggesting that the tHcy effect on blood glucose level/GDM is independent of BMI, B_12_ and folate (ESM Table 5).

#### Low B_12_–high folate imbalance on blood glucose level and GDM

While no multiplicative interactions were observed between B_12_ and folate as continuous variables (interaction effects on FPG, 2 h-PG, NICE-GDM and IADPSG-GDM having *p* values of 0.47, 0.20, 0.83 and 0.49, respectively), significant interactions were observed when B_12_ and folate were categorised as tertiles with RR of NICE-GDM (ESM Table 6). The opposing associations of B_12_ and folate with glucose and GDM result in the highest risk being in women within the lowest B_12_ and highest folate tertiles (ESM Fig. 2a–d). Compared with the ‘B_12_ tertile3+folate tertile1’ group, the ‘B_12_ tertile1+folate tertile3’ group had higher levels of FPG (0.21 mmol/l; *p* = 0.0001) and 2 h-PG (0.57 mmol/l; *p* < 0.0001) and higher aRR of IADPSG-GDM (aRR 1.742; 95% CI 1.226, 2.437; *p* = 0.003).

#### Ethnic differences of B_12_, folate and tHcy in blood glucose level and GDM risk

The B_12_ associations seemed to be stronger in South Asians, with a 22% lower RR for IADPSG-GDM in model 1 (aRR 0.78; 95% CI 0.63, 0.947; *p* = 0.01) and 14.7% in model 2 (aRR 0.853; 95% CI 0.689, 1.037; *p* = 0.12) (Fig. 4a). The inverse association with FPG was seen in all ethnic groups, but, for 2h-PG, it was significant only in South Asians in model 2 (Fig. 3a,b). However, no significant differences were observed between ethnic groups when an interaction function was used between B_12_ and ethnicity. The folate association with 2h-PG seemed to be present in all ethnic groups, but, for the risk of GDM, it was statistically significant only in white people (Figs 3d, 4b). The tHcy associations with FPG, 2h-PG and GDM were present only in white people (data not shown).

## Discussion

In this early pregnancy prospective cohort study, we found that B_12_ insufficiency and folate excess were common in the first trimester. B_12_ levels were lower and folate levels were higher in women who were subsequently diagnosed to have GDM, around 27 weeks of gestation. Lower B_12_ in early pregnancy was associated with higher fasting and 2 h glucose levels as well as a higher RR of GDM. Folate associations were opposite, and higher folate level was associated with higher 2h-PG and higher risk of GDM. Approximately half of the effect sizes were mediated by BMI. These associations of B_12_ and folate levels with maternal blood glucose level were clinically small (between 0.06 and 0.08 mmol/l per SD) but statistically significant. Although no interactions were observed for B_12_ and folate levels (as a continuum) with glucose and GDM, a combination of low B_12_ and high folate tertiles had stronger associations with hyperglycaemia and GDM than the individual B_12_/folate levels. Having a larger sample size with multiple ethnic groups enabled us to explore the complex associations of folate with FPG and 2h-PG, and to clarify previously inconsistent findings with greater confidence.

Higher FPG and risk of GDM at low B_12_ levels was present in all ethnic groups, but the inverse association with 2h-PG was present mainly in South Asians. Similar inverse relationships at the time of OGTT were observed for B_12_ with FPG and insulin resistance in White British women [16], and with GDM in Indians (living in India and Singapore) [14, 15] and Italians [25]. In contrast, a recent study showed a direct relationship between early pregnancy B_12_ and 1 h and 2 h plasma glucose levels in a Chinese population. However, this study did not report its association with FPG or the relationship after adjusting for BMI or other covariates [21]. In addition, it did not report the association between B_12_ and BMI. These factors may have accounted for the differences, especially due to the inverse relationship observed between B_12_ and BMI in our study. This inverse relationship between B_12_ and BMI has been seen in other studies both during [10, 16, 17] and outside pregnancy [38].

Two recent Mendelian randomisation studies, primarily in white people, did not demonstrate a causal link between B_12_ and BMI [39, 40]. *FUT2* polymorphisms can influence the composition of gut microbes [41] which in turn can cause both obesity and low B_12_. This was proposed as a potential explanation for the strong observational link but lack of Mendelian randomisation link between low B_12_ and high BMI [40]. Our in vitro studies showed that subcutaneous adipose tissue isolated at the time of delivery from pregnant women with low B_12_ levels had increased expression of adipogenic and lipogenic genes, as well as altered expression of 12 microRNAs (miRNAs) that regulate the peroxisome proliferator-activated receptor (PPAR)γ and insulin signalling pathways [38, 42]. These adipose tissue-derived miRNAs were also altered in the maternal circulation [42], which in turn can affect the beta cell function and/or hepatic handling of glucose levels [43, 44].

Our study provides clarification for conflicting data that have been reported recently on maternal folate levels and GDM risk. Our large sample size enabled us to demonstrate a U-shaped relationship between folate and FPG levels and may explain the opposite associations reported in different populations. High folate levels within the reference range may be beneficial for FPG but harmful for 2 h-PG, and folate levels above the reference range appear to be harmful for both. The relationship of higher folate and higher 2h-PG with GDM risk is similar to observations reported in Chinese [19–21] and Indian populations [15] but opposite to that in the American population [18], which only had estimated folate intake. The American study was conducted prior to mandatory folate fortification and hence was unlikely to have had the high levels of folate observed in our population. It has been shown that excess folic acid from supplements results in the presence of unmetabolised folic acid levels in serum in all age groups [45]. Unmetabolised folic acid slows the 1-C cycle, reduces the methylation potential and conversion of methionine, and increases intracellular accumulation of ATP [46]. This can lead to reduced insulin-mediated glucose uptake in muscle cells in vitro and in animal models, resulting in reduced insulin sensitivity and higher insulin resistance in the offspring [45, 46]. This may explain the opposite associations we observed for folate levels within the reference range with FPG and 2h-PG levels. In addition, a ‘low B_12_–high folate’ combination could have compromised the methylation potential further, which is known to be associated with adverse metabolic risk [47, 48].

tHcy had inverse associations with glucose levels and adjusting for BMI augmented its effect size on blood glucose level. Similar inverse associations between tHcy and glucose levels were also observed recently [15]. We found that higher BMI is associated with higher tHcy and glucose levels. In addition, it is known that high tHcy levels can be associated with low birthweight [49], and 5–7% of offspring of women with GDM may be small for gestational age [2, 3]. Further investigations are warranted to understand the complex relationships among tHcy, glucose levels and birthweight.

### Strengths and limitations

The strengths of our study were that it was a large, early pregnancy, multi-ethnic, prospective cohort in a UK representative population, with more than 90% follow-up. We centrifuged our samples within 30 min, and measured folate up to 136 nmol/l and tHcy by the LCMS method. However, our study had the following limitations. First, we did not have glucose measurements in early pregnancy or 30 min or 1 h glucose and insulin measurements at OGTT to further explore the associations of B_12_/folate with beta cell function or insulin resistance. Lack of 1 h glucose levels may have underestimated the IADPSG-GDM rates. However, we believe this is unlikely to have influenced our conclusions, as the observed associations between these B vitamins and glucose levels were continuous. Second, we did not have oxidised folate (or sub-fractions of folate) measurements. While this is unlikely to have influenced our results, it may have helped to explore the relationship between unmetabolised folic acid and 2 h-PG. Third, the tHcy levels observed seem to be higher than in other studies. Our first trimester samples were non-fasted samples. Food is known to increase the tHcy levels [30]. In addition, our sensitive, LCMS-based assay could have contributed to these higher levels [50]. Fourth, as our study used NICE selective screening criteria, two-thirds of white women were screened because of obesity, and hence our findings may not be applicable to normal weight white women. Finally, although our cohort was relatively large, for our ethnic-specific analyses, it was still small, as shown by the wide CIs.

### Implications, unanswered questions and future research

Our study demonstrated that B_12_ and folate levels may be independent, modifiable risk factors for hyperglycaemia in late pregnancy. B_12_ insufficiency is not uncommon in pregnancies across the world [51]. Our study confirmed this and showed that excess folate levels are also common, while folate deficiency was rare in early pregnancy, highlighting the need to avoid very high folate levels in early pregnancy. This warrants urgent review of the dose and duration of folic acid supplements and future research should focus on: (1) investigating the effect of optimising B_12_ and folate levels in early pregnancy and before conception on glucose levels and rate of GDM and/or its complications; (2) mechanistic studies investigating the effects of food- vs supplement-derived folate and unmetabolised folic acid levels on glucose levels and their potential epigenetic effects; and (3) studies designed to explore the complex relationships among tHcy, BMI, glucose and birthweight.

## Supplementary Information

ESM 1 (PDF 544 kb)

## Data Availability

Individual participant data that underlie the results reported in this article will be available from 9 to 36 months following the publication of this article. The data will be shared with researchers who provide a methodologically sound proposal that has been approved by an independent review committee to achieve the aims described in their proposal. Proposals should be directed to p.saravanan@warwick.ac.uk and Y.Weldeselassie@warwick.ac.uk to gain access. A data access agreement must be signed prior to access as per University of Warwick standard operating procedures.

## Abbreviations

aRR: Adjusted RR
BCAA: Branched-chain amino acid
1-C: Single carbon atoms
FPG: Fasting plasma glucose
GDM: Gestational diabetes mellitus
2 h-PG: 2 h post-load plasma glucose
IADPSG: International Association of Diabetes and Pregnancy Study Groups
IADPSG-GDM: GDM diagnosis based on IADPSG criteria
LCMS: Liquid chromatography mass spectrometry
miRNA: MicroRNA
NICE: National Institute for Health and Care Excellence
NICE-GDM: GDM diagnosis based on NICE criteria
SES: Socioeconomic status
tHcy: Total homocysteine
