# Supplementary material for: Association of maternal vitamin B12 and folate levels in early pregnancy with gestational diabetes: a prospective UK cohort study (PRiDE study)
Source: Diabetologia. 2021 Jul 22;64(10):2170–82. doi: 10.1007/s00125-021-05510-7 (PMC8423653; doi:10.1007/s00125-021-05510-7)
Supplement: Supplementary file 1 — (PDF 544 kb) [file 125_2021_5510_MOESM1_ESM.pdf]

**Electronic Supplementary Material**

**Association of maternal vitamin B12 and folate levels in early pregnancy with gestational diabetes: a prospective UK cohort study (PRiDE study)**

Ponnusamy Saravanan, Nithya Sukumar, Antonysunil Adaikalakoteswari, Ilona Goljan, Hema Venkataraman, Amitha Gopinath, Christos Bagias, Chittaranjan S Yajnik, Nigel Stallard, Yonas Ghebremichael-Weldeselassie, Caroline HD Fall

**Table of contents**

Electronic supplementary tables 2-7

ESM Table 1: ESM Table 1: Predictors of B12, Folate and tHcy 2

ESM Table 2: Reasons for OGTT 3

ESM Table 3: Characteristics of study participants by NICE GDM 4

ESM Table 4: B12 insufficiency and risk of GDM 5

ESM Table 4: Associations of tHcy with glycaemia and risk of GDM 6

ESM Table 6: Interaction between B12 and folate tertiles with glycaemia and GDM 7

Electronic supplementary figures 8-12

ESM Figure 1a-b: Early pregnancy B12/folate levels and risk of NICE GDM 8-9

ESM Figure 2a-d: Associations of early pregnancy 'low B12 and high folate' with fasting and 2hr-glucose levels and risk of GDM 10-12

**Electronic Supplementary Materials - Tables**

**ESM Table 1: Predictors of B12, Folate and tHcy**

| | **B12**<br>**β (95% CI)** | **p-value** | **Folate**<br>**β (95% CI)** | **p-value** | **tHcy**<br>**β (95% CI)** | **p-value** |
|---|---|---|---|---|---|---|
| B12§ | NA | NA | 1.96 (1.1, 2.83) | <0.0001 | -0.85 (-1.05, -0.66) | <0.0001 |
| Folate§ | 7.91 (4.42, 11.40) | <0.0001 | NA | NA | -0.85 (-1.04, -0.65) | <0.0001 |
| **Supplements** | | | | | | |
| Folic Acid | -4.27 (-12.47, 3.93) | 0.31 | 13.51 (11.56,15.46) | <0.0001 | -0.75 (-1.20, -0.29) | 0.001 |
| Multivitamin | 32.15 (25.16, 39.15) | <0.0001 | 0.31 (-1.40, 2.03) | 0.72 | -0.66 (-1.05,-0.27) | 0.0009 |
| Age§ | 6.34 (2.64, 10.04) | 0.0008 | 4.19 (3.30, 5.08) | <0.0001 | -0.30 (-0.50, -0.09) | 0.005 |
| BMI§ | -21.30 (-24.97, -17.62) | <0.0001 | 2.20 (1.29, 3.10) | <0.0001 | 0.42 (0.21, 0.63) | 0.0001 |
| Multi parity (≥2) | -3.8284 (-12.73, 5.08) | 0.40 | -6.79 (-8.94, -4.64) | <0.0001 | 0.50 (0.0, 0.99) | 0.05 |
| **Ethnicity (ref: White people)** | | | | | | |
| Others | 96.48 (84.87, 108.09) | <0.0001 | -1.28 (-4.19, 1.64) | 0.39 | -0.22 (-0.88, 0.45) | 0.52 |
| South Asians | -12.59 (-22.89, -2.29) | 0.02 | 3.96 (1.46, 6.46) | 0.002 | 0.13 (-0.44, 0.70) | 0.65 |
| **Smoking (ref: never smoked)** | | | | | | |
| Stopped during pregnancy | -3.59 (-16.49, 9.31) | 0.59 | -0.44 (-3.57, 2.69) | 0.78 | 0.24 (-0.47, 0.95) | 0.51 |
| Stopped pre pregnancy | 2.58 (-6.63, 11.78) | 0.58 | 0.06 (-2.18, 2.29) | 0.96 | -0.64 (-1.15, -0.13) | 0.01 |
| Continuing to smoke | -0.02 (-11.44, 11.40) | 1.00 | -6.00 (-8.77, -3.24) | <0.0001 | 1.12 (0.49, 1.74) | 0.0005 |
| **Household income**<br>**(ref: £20,800 up to £31,199)** | | | | | | |
| <£10,400 | -6.69 (-19.77, 6.39) | 0.32 | 0.003 (-3.17, 3.18) | 1.00 | 1.14 (0.42, 1.86) | 0.002 |
| £10,400 up to £20,799 | -8.80 (-21.01, 3.41) | 0.16 | 1.55 (-1.4, 4.52) | 0.30 | 0.54 (-0.13, 1.21) | 0.12 |
| £31,200 up to £51,999 | 1.24 (-10.36, 12.85) | 0.83 | 2.68 (-0.14, 5.50) | 0.06 | 0.41 (-0.23, 1.05) | 0.21 |
| £52,000 and above | 7.0015 (-5.09, 19.09) | 0.26 | 3.30 (0.37, 6.24) | 0.03 | 0.36 (-0.31, 1.02) | 0.29 |
| Prefer not to say | -10.44 (-22.38, 1.50) | 0.09 | 0.31 (-2.59, 3.21) | 0.83 | 1.09 (0.43, 1.74) | 0.001 |

§Standardised β for likewise comparisons
Abbreviations: BMI- Body Mass Index; CI- Confidence Interval; tHcy- total Homocysteine

**ESM Table 2: Reasons for OGTT**

| | **All (n=4743) n (%)** | **White people (n=3487) n (%)** | **South Asians (n=766) n (%)** | **Others (n=489) n (%)** |
|---|---|---|---|---|
| BMI ≥30 | 2656 (56.0) | 2376 (68.1) | 115 (15.0) | 165 (33.7) |
| Previous GDM | 265 (5.6) | 189 (5.4) | 50 (6.5) | 26 (5.3) |
| Family history of Diabetes | 2004 (42.3) | 1449 (41.6) | 376 (49.1) | 179 (36.6) |
| Ethnic minority origin | 1256 (26.5) | NA | NA | NA |

Abbreviations: BMI- Body Mass Index; GDM- Gestational Diabetes Mellitus; OGTT- Oral Glucose Tolerance Test

**ESM Table 3: Characteristics of study participants by NICE GDM**

| **Maternal Characteristics** | **All (n=4320)<br>Mean ± SD<br>Median (IQR)[§]<br>n (%)^** | **GDM (n=538)<br>Mean ± SD<br>Median (IQR)[§]<br>n (%)^** | **Non GDM (n=378)<br>Mean ± SD<br>Median (IQR)[§]<br>n (%)^** | **p-value** |
|---|---|---|---|---|
| **Ethnicity^** | | | | |
| White people | 3188 (73.8) | 390 (12.2) | 2798 (87.8) | 0.59 |
| South Asians | 697 (16.1) | 95 (13.6) | 602 (86.4) | .. |
| Others | 435 (10.1) | 53 (12.2) | 382 (88.8) | .. |
| Age (years) | 30.59 ± 5.23 | 32.35 ± 5.3 | 30.34 ± 5.17 | <0.0001 |
| Multi parity (≥2)^ | 806 (18.7) | 97 (18) | 709 (18.7) | 0.73 |
| Visit 1 gestational age (weeks) | 12.47 ± 1.41 | 12.41 ± 1.46 | 12.48 ± 1.4 | 0.34 |
| Gestational age at OGTT (weeks)[a] | 26.79 ± 2.64 | 25.8 ± 4.05 | 26.93 ± 2.35 | <0.0001 |
| Height (cm) | 164.33 ± 6.85 | 163.51 ± 6.92 | 164.45 ± 6.83 | 0.003 |
| Weight (kg) | 83.54 ± 20.46 | 87.01 ± 20.19 | 83.05 ± 20.45 | <0.0001 |
| BMI ($kg/m^2$) | 30.86 ± 7.07 | 32.44 ± 6.81 | 30.64 ± 7.07 | <0.0001 |
| Waist circumference (cm)[b] | 98.57 ± 16.35 | 102.4 ± 15.98 | 98.02 ± 16.33 | <0.0001 |
| Gestational weight gain (kg)[c] | 5.86 ± 4.75 | 5.18 ± 4.77 | 5.95 ± 4.74 | 0.001 |
| Using Folate supplements^ | 3136 (78.5) | 406 (81.4) | 2729 (78.1) | 0.11 |
| Using Multivitamin supplements^ | 2317 (59.2) | 295 (61.2) | 2022 (59.0) | 0.37 |
| **Biochemical Characteristics** | **n=4228** | **n=526** | **n=3702** | |
| B12 (pmol/l)[§] | 239.1 (183.76, 313.3) | 230.5 (183.0, 307.3) | 240.5 (184.2, 314.1) | 0.05 |
| Folate (nmol/l)[§] | 36.7 (25.5, 52.7) | 40.7 (28.2, 57.9) | 36.1 (25.1, 52.2) | 0.001 |
| tHcy (µmol/l)[§d] | 11.2 (8.6, 14.6) | 10.6 (8.0, 13.9) | 11.4 (8.7, 14.7) | 0.0006 |
| B12 insufficiency at <150pmol/l^ | 436 (10.3) | 56 (10.6) | 380 (10.3) | 0.85 |
| B12 insufficiency at <220pmol/l^ | 1790 (42.3) | 237 (45.1) | 1553 (42.0) | 0.19 |
| Folate deficiency (<10nmol/l)^ | 54 (1.3) | 6 (1.1) | 48 (1.3) | 0.93 |
| Folate excess (>45nmol/l)^ | 1544 (36.5) | 215 (40.9) | 1329 (35.9) | 0.03 |

Variables that have lesser 'n' are indicated separately:
[a]All = 4227, GDM = 513, Non GDM = 3714;
[b]All = 3979, GDM = 499, non-GDM = 3480;
[c]All = 3968, GDM = 473, non-GDM = 3495;
[d]All = 4209, GDM = 519, non-GDM = 3690.
Abbreviations: SD- Standard Deviation; IQR- Interquartile Range; BMI- Body Mass Index; GDM- Gestational Diabetes Mellitus; NICE- The National Institute for Health and Care Excellence; OGTT- The Oral Glucose Tolerance Test; tHcy- total Homocysteine

**ESM Table 4: B12 insufficiency and risk of GDM**

| | NICE GDM | | IADPSG GDM | |
|---|---|---|---|---|
| | **aRR (95% CI)** | **p-value** | **aRR (95% CI)** | **p-value** |
| **B12 insufficiency at <150pmol/l** | | | | |
| **Model 1** | | | | |
| All ethnic groups combined | 1.135(0.837,1.539) | 0.42 | 1.221(0.926,1.609) | 0.16 |
| White people | 0.955(0.659,1.384) | 0.81 | 1.095(0.785,1.527) | 0.59 |
| South Asians | 1.367(0.731,2.558) | 0.33 | 1.427(0.818,2.491) | 0.21 |
| Others | 3.781(1.331,10.74) | 0.01 | 2.547(0.864,7.509) | 0.09 |
| **Model 2** | | | | |
| All ethnic groups combined | 1.018(0.747,1.386) | 0.91 | 1.06(0.799,1.407) | 0.68 |
| White people | 0.843(0.578,1.227) | 0.37 | 0.932(0.664,1.31) | 0.69 |
| South Asians | 1.265(0.672,2.379) | 0.47 | 1.285(0.73,2.263) | 0.38 |
| Others | 3.836(1.311,11.225) | 0.01 | 2.545(0.834,7.767) | 0.1 |
| **B12 insufficiency at <220pmol/l** | | | | |
| **Model 1** | | | | |
| All ethnic groups combined | 1.235(1.02,1.496) | 0.03 | 1.383(1.157,1.652) | 0.0004 |
| White people | 1.081(0.866,1.35) | 0.49 | 1.178(0.957,1.45) | 0.12 |
| South Asians | 1.226(0.789,1.906) | 0.36 | 1.755(1.183,2.605) | 0.005 |
| Others | 4.115(2.2,7.698) | <0.0001 | 3.356(1.822,6.181) | 0.0001 |
| **Model 2** | | | | |
| All ethnic groups combined | 1.102(0.907,1.34) | 0.33 | 1.203(1.003,1.443) | 0.05 |
| White people | 0.953(0.76,1.195) | 0.68 | 1.009(0.816,1.248) | 0.93 |
| South Asians | 1.142(0.732,1.78) | 0.56 | 1.61(1.08,2.401) | 0.02 |
| Others | 3.763(1.993,7.105) | <0.0001 | 2.971(1.589,5.554) | 0.001 |

Model 1: Age, parity, family history, household income, smoking and folate
Model 2: Model 1 + BMI
Abbreviations: aRR- adjusted Relative Risk; CI- Confidence Interval; GDM- Gestational Diabetes Mellitus; NICE- The National Institute for Health and Care Excellence; IADPSG- The International Association of Diabetes and Pregnancy Study Groups

**ESM Table 5: Associations of tHcy with glycaemia and risk of GDM**

| | Standardised β (95% CI) | p-value |
|---|---|---|
| | **Fasting Glucose (mmol/l)** | |
| Model 1 | -0.025 (-0.044, -0.006) | 0.01 |
| Model 2 | -0.034 (-0.053, -0.016) | 0.0002 |
| | **2-h Glucose (mmol/l)** | |
| Model 1 | -0.043 (-0.095, 0.009) | 0.1 |
| Model 2 | -0.058 (-0.109, -0.007) | 0.03 |
| | **NICE GDM** | |
| | **aRR (95% CI)** | **p-value** |
| Model 1 | 0.808 (0.711, 0.918) | 0.001 |
| Model 2 | 0.779 (0.684, 0.888) | 0.0002 |
| | **IADPSG GDM** | |
| | **aRR (95% CI)** | **p-value** |
| Model 1 | 0.848 (0.757, 0.95) | 0.005 |
| Model 2 | 0.811 (0.722, 0.912) | 0.0005 |

Model 1: Age, parity, family history, ethnicity, household income, smoking, B12 and Folate
Model 2: Model 1 + BMI
tHcy values are standardised
Abbreviations: aRR- adjusted Relative Risk; CI- Confidence Interval; GDM- Gestational Diabetes Mellitus; NICE- The National Institute for Health and Care Excellence; IADPSG- The International Association of Diabetes and Pregnancy Study Groups; tHcy- total Homocysteine

**ESM Table 6: Interaction of B12 and folate in tertiles with glycaemia and GDM**

| | **B12 tertile 1** | | | | **B12 Tertile 2** | | | |
|---|---|---|---|---|---|---|---|---|
| | **FPG (β; 95% CI)** | **2hr-PG (β; 95% CI)** | **NICE GDM** | **IADPSG GDM** | **FPG (β; 95% CI)** | **2hr-PG (β; 95% CI)** | **NICE GDM** | **IADPSG GDM** |
| Folate Tertile 2 | -0.05 (-0.15, 0.05) | 0.16 (-0.11, 0.44) | **0.48 (0.25, 0.86)** | 0.96 (0.58, 1.52) | -0.01 (-0.11, 0.08) | -0.1 (-0.38, 0.18) | **0.52 (0.28, 0.94)** | 0.94 (0.56, 1.51) |
| | p=0.34 | p=0.24 | **p=0.02** | p=0.88 | p=0.79 | p=0.49 | **p=0.03** | p=0.81 |
| Folate Tertile 3 | -0.03 (-0.12, 0.07) | 0.16 (-0.11, 0.44) | 0.8 (0.44, 1.37) | 1.18 (0.73, 1.8) | -0.05 (-0.14, 0.05) | -0.05 (-0.32, 0.23) | **0.49 (0.26, 0.89)** | 0.95 (0.56, 1.51) |
| | p=0.58 | p=0.25 | p=0.43 | p=0.48 | p=0.35 | p=0.75 | **p=0.02** | p=0.82 |

Values shown are β coefficient or aRR (95%CI) and p-value for interactions with various combinations of B12 and Folate tertiles; Reference tertiles: B12 tertile 3 and Folate tertile 1

Covariates in the model: Age, parity, ethnicity, family history, household income, smoking and BMI
Abbreviations: FPG- Fasting Plasma Glucose; 2hr-PG- 2 hours Plasma Glucose; CI- Confidence Interval; GDM- Gestational Diabetes Mellitus; NICE- The National Institute for Health and Care Excellence; IADPSG- The International Association of Diabetes and Pregnancy Study Groups; BMI- Body Mass Index

**Electronic Supplementary Material - Figures:**

**ESM Fig. 1a-b: Early pregnancy B12/folate levels and risk of NICE GDM**

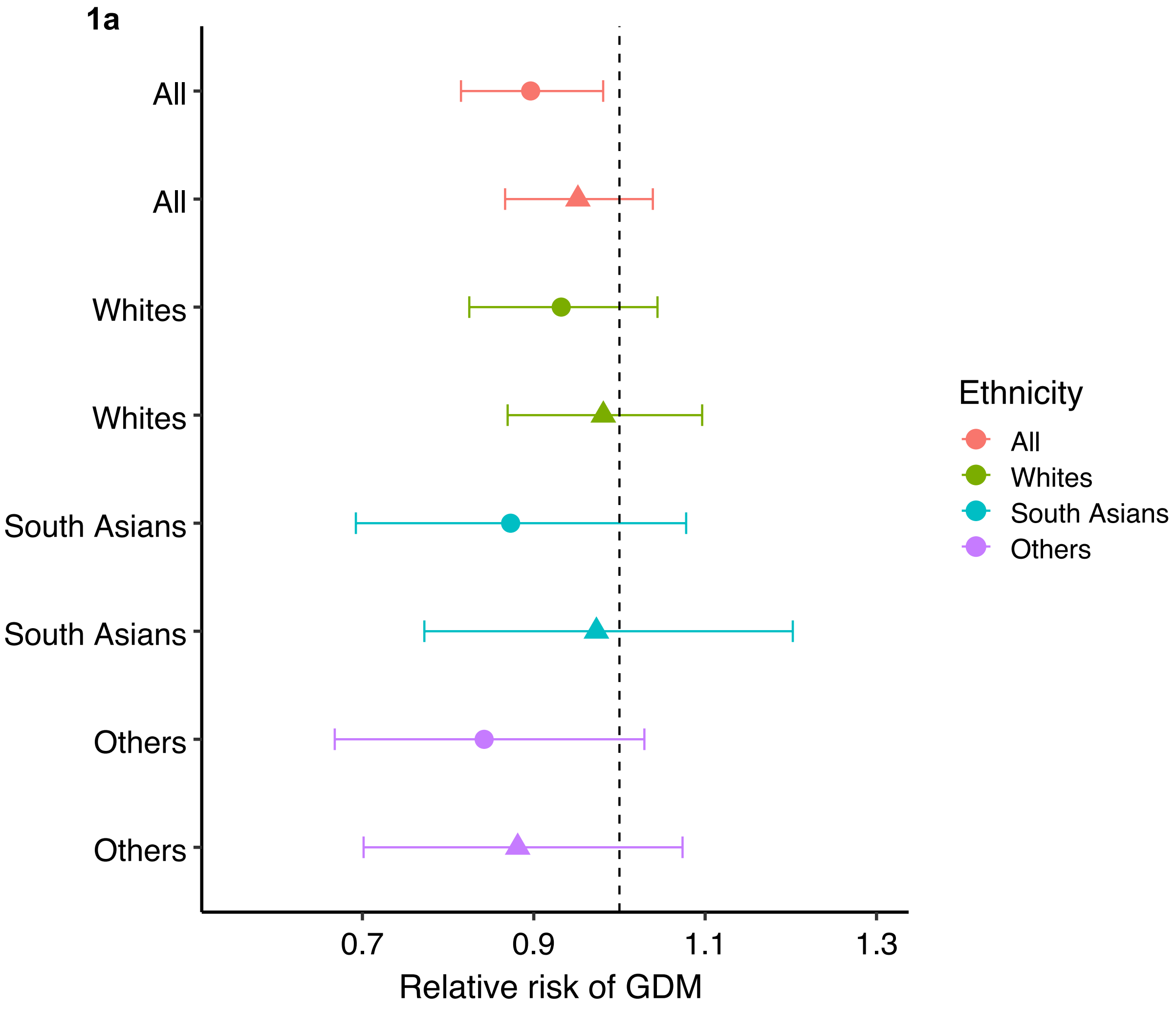

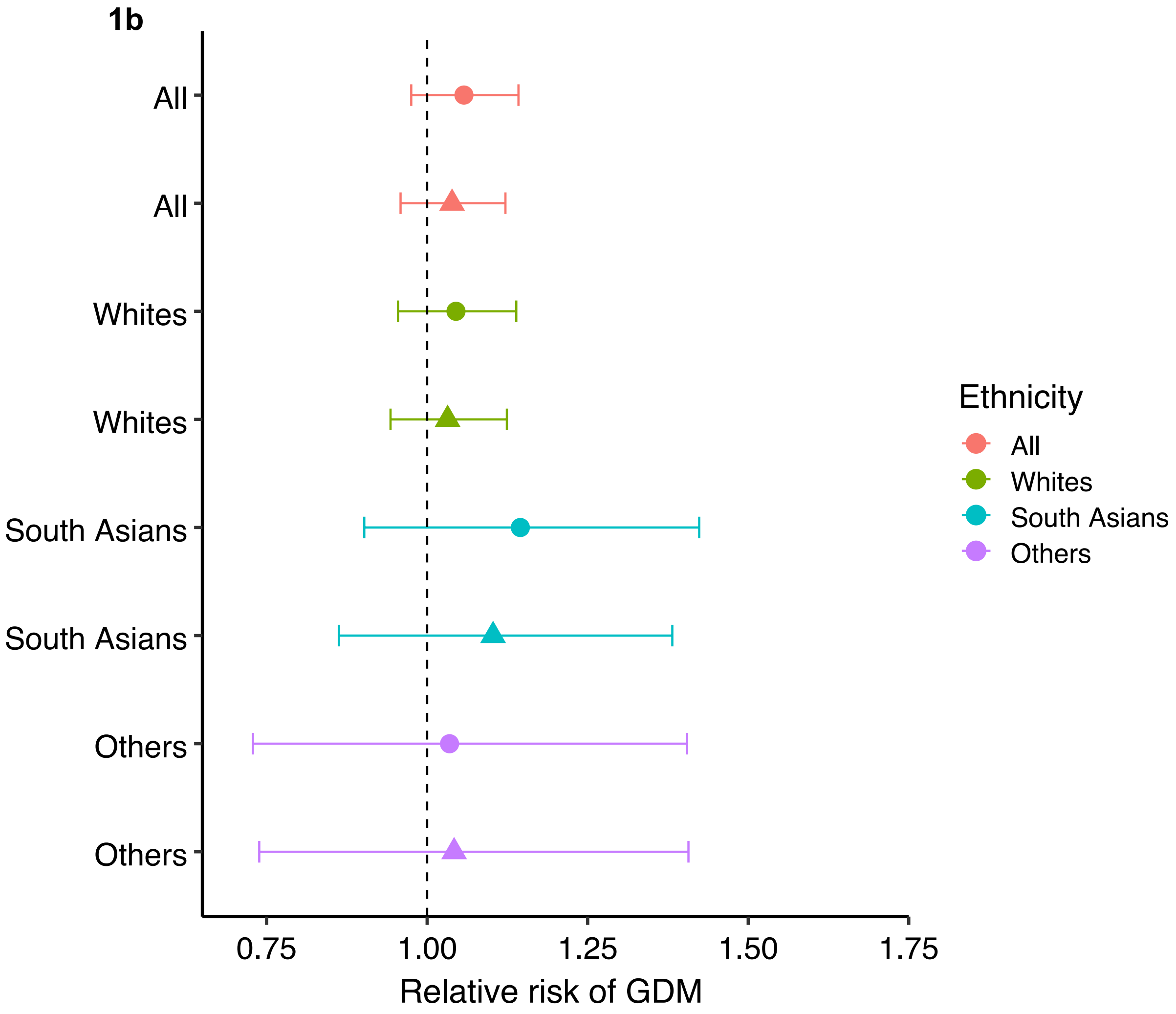


Panel ‘a’ shows the B12 and ‘b’ shows the folate levels in early pregnancy and adjusted relative risk of NICE GDM. All participants are shown in red, White people in green, South Asians in blue, and Others in purple lines with 95% CI whiskers. Two models are shown for each ethnic group. Closed circle depicts model 1 and triangle depicts model 2. Model 1 is adjusted for the following co-variates: Age, parity, family history, household income, smoking and B12. Model 2 is adjusted for model 1 + BMI.

**ESM Fig. 2a-d: Associations of early pregnancy 'low B12 and high folate' with fasting and 2hr-glucose levels and risk of GDM**

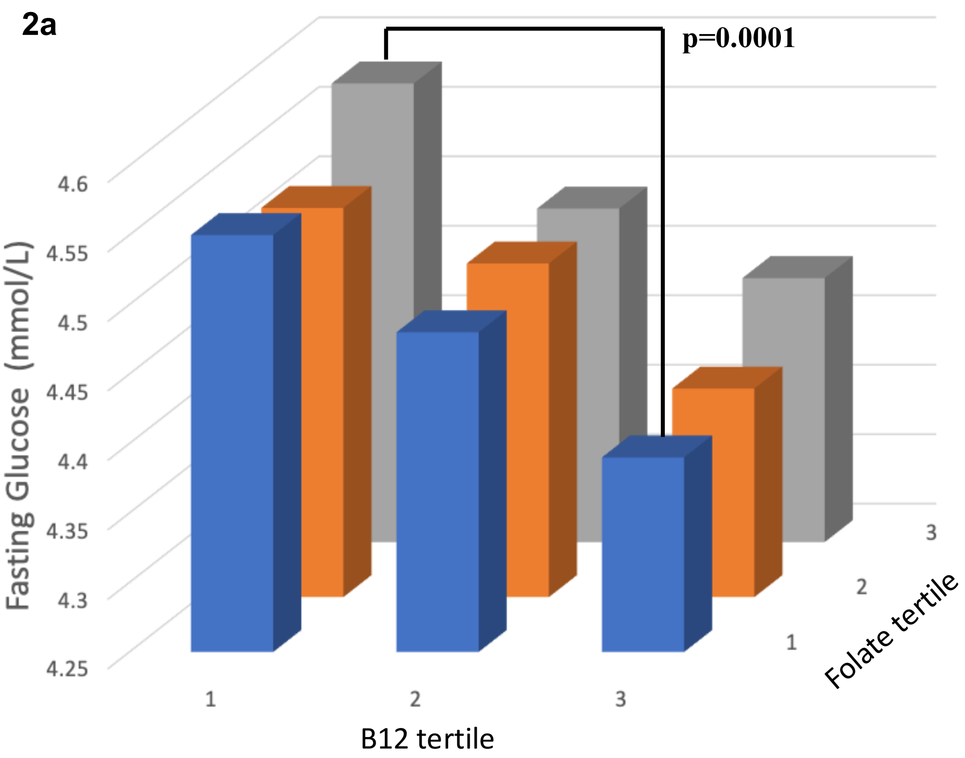


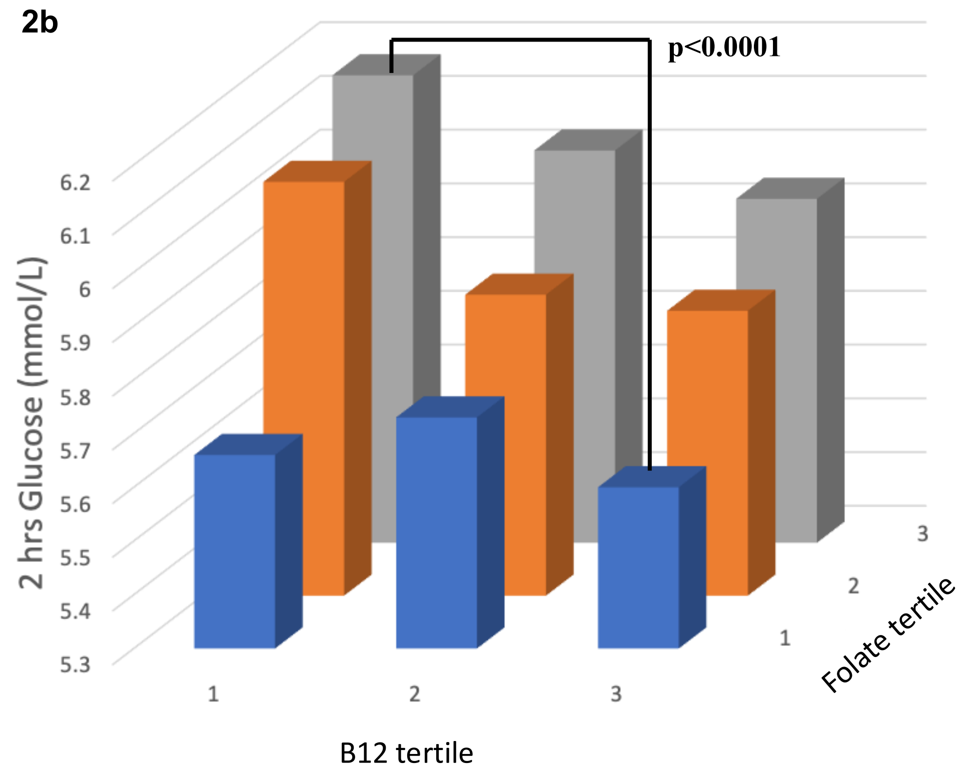

**2c**

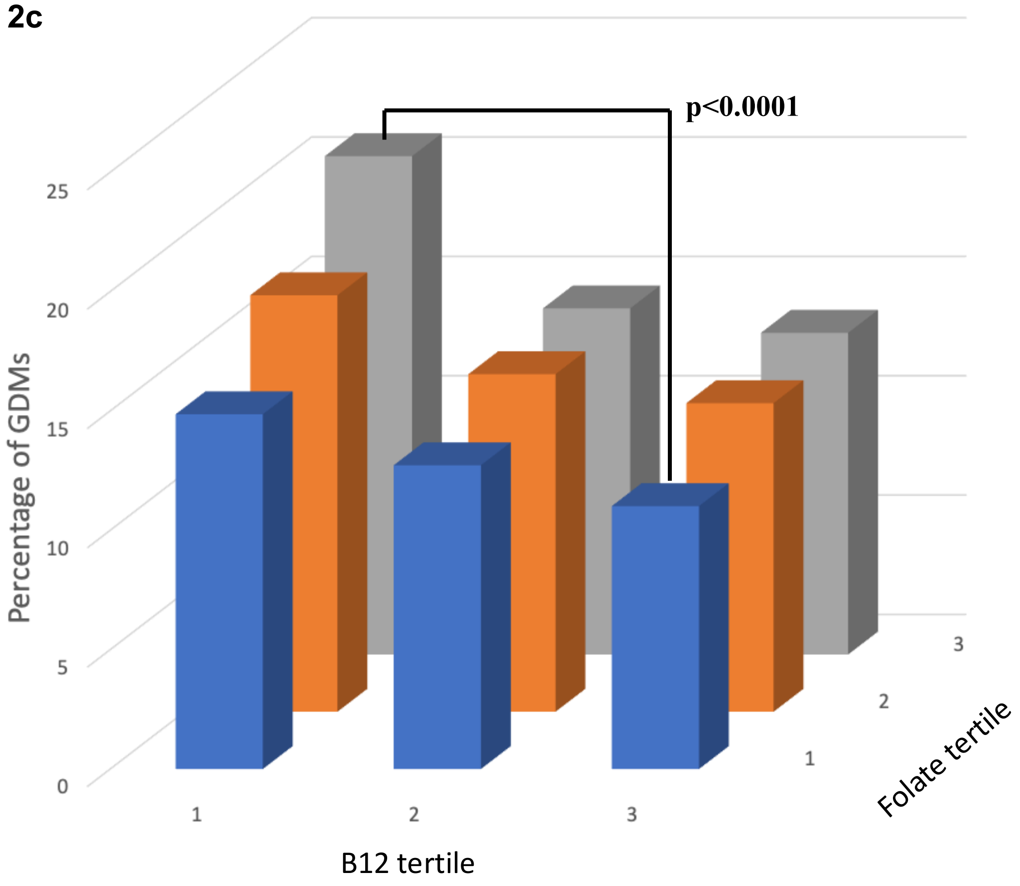

p<0.0001
25
20
15
10
5
0
Percentage of GDMs
1
2
3
B12 tertile
1
2
3
Folate tertile


**2d**

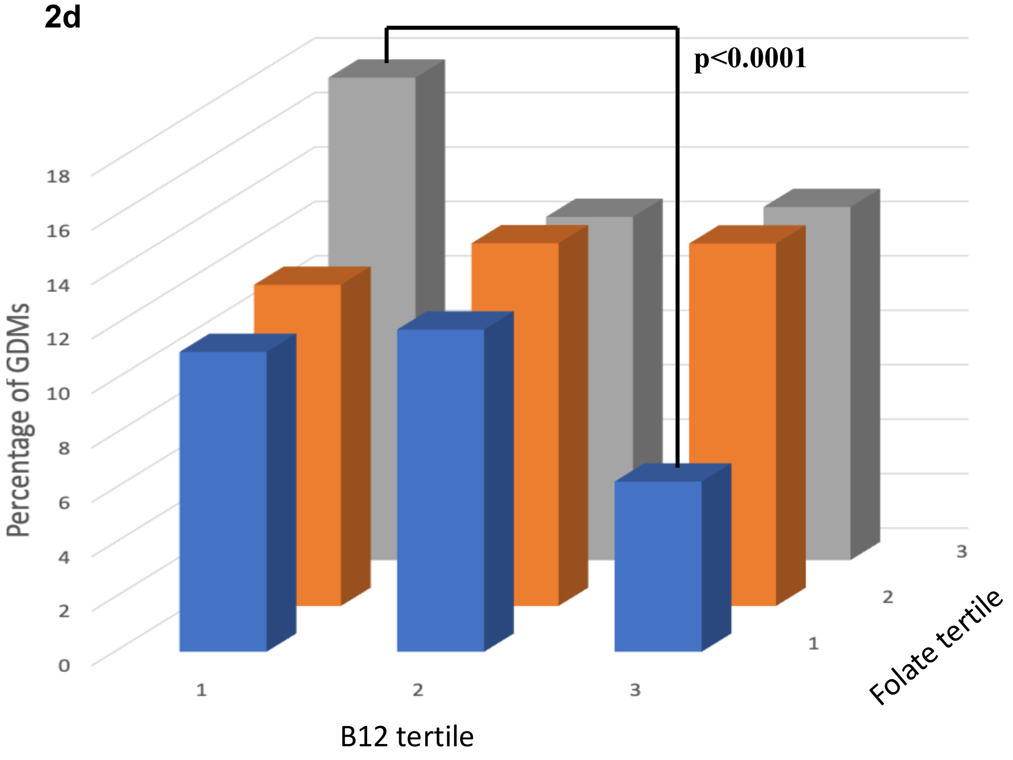

p<0.0001
18
16
14
12
10
8
6
4
2
0
Percentage of GDMs
1
2
3
B12 tertile
1
2
3
Folate tertile

Panel ‘a’ shows the associations of early pregnancy combined B12 and folate tertiles with fasting glucose at OGTT, and panel ‘b’ with 2hr-glucose levels. Panel ‘c’ shows the proportions of GDM by IADPSG and ‘d’ by NICE definitions in each of the combinations. ‘p’ value shown in figure is only for the difference between B12 tertile1+folate tertile 3 vs. B12 tertile 3+folate tertile 1.
